# Detection of Limbal Stem Cells Adhered to Melt Electrospun Silk Fibroin and Gelatin-Modified Polylactic Acid Scaffolds

**DOI:** 10.3390/polym15030777

**Published:** 2023-02-03

**Authors:** Emilija Zdraveva, Krešo Bendelja, Luka Bočkor, Tamara Dolenec, Budimir Mijović

**Affiliations:** 1 Department of Fundamental Natural and Engineering Sciences, Faculty of Textile Technology, University of Zagreb, 10000 Zagreb, Croatia; 2 Center for Research and Knowledge Transfer in Biotechnology, University of Zagreb, 10000 Zagreb, Croatia; 3 Center for Applied Bioanthropology, Institute for Anthropological Research, 10000 Zagreb, Croatia; 4 Department of Transfusion and Regenerative Medicine, Sestre Milosrdnice University Hospital Center, 10000 Zagreb, Croatia

**Keywords:** melt electrospinning, PLA/silk fibroin, PLA/gelatin, LSCs, number of viable cells, immunocytochemistry

## Abstract

Limbal stem cells (LSCs) are of paramount importance in corneal epithelial tissue repair. The cornea becomes opaque in case of limbal stem cell deficiency (LSCD), which may cause serious damage to the ocular visual function. There are many techniques to restore damaged epithelium, one of which is the transplantation of healthy cultured LSCs, usually onto a human amniotic membrane or onto bio-based engineered scaffolds in recent years. In this study, melt electrospun polylactic acid (PLA) was modified by silk fibroin or gelatin and further cultured with LSCs originating from three different donors. In terms of physicochemical properties, both modifications slightly increased PLA scaffold porosity (with a significantly larger pore area for the PLA/gelatin) and improved the scaffolds’ swelling percentage, as well as their biodegradation rate. In terms of the scaffold application function, the aim was to detect/visualize whether LSCs adhered to the scaffolds and to further determine cell viability (total number), as well as to observe p63 and CK3 expressions in the LSCs. LSCs were attached to the surface of microfibers, showing flattened conformations or 3D spheres in the formation of colonies or agglomerations, respectively. All scaffolds showed the ability to bind the cells onto the surface of individual microfibers (PLA and PLA/gelatin), or in between the microfibers (PLA/silk fibroin), with the latter showing the most intense red fluorescence of the stained cells. All scaffolds proved to be biocompatible, while the PLA/silk fibroin scaffolds showed the highest 98% viability of 2.9 × 10^6^ LSCs, with more than 98% of p63 and less than 20% of CK3 expressions in the LSCs, thus confirming the support of their growth, proliferation and corneal epithelial differentiation. The results show the potential of these bio-engineered scaffolds to be used as an alternative clinical approach.

## 1. Introduction

After the brain, the eye is the second most complex human organ. Its front surface is the transparent, curved cornea that serves as a protective barrier of the eye from the environment, and it consists of three layers: the outer epithelium, the middle stroma, and the inner endothelium [1]. The small size (1–2 mm) limbus is the zone with no precise border lines that separates the optically clear cornea, the conjunctiva, the opaque sclera, and the uvea. The pathways of the aqueous humor outflow, the trabecular meshwork, Schlemm’s canal, and the aqueous collector channels are located in the limbus, which also provides access incisions for cataract and glaucoma surgery [2]. The World Health Organization reports at least 2.2 billion people with visual impairment, of which 88.4 million are blind due to unaddressed refractive error. Causes of visual impairment include age-related macular degeneration, cataract, diabetic retinopathy, glaucoma, and uncorrected refractive errors [3]. The limbus is the reservoir of corneal epithelial stem cells [4]; the corneal epithelial tissue repair and regeneration is reportedly enabled through the activity of the so-called limbal stem cells (LCSs) situated in the limbus. The limbal stem cells have the ability to renew themselves and produce new cells that differentiate progressively [5,6]. LSC failure or deficiency (cells population depletion or dysfunction of the LCS stromal microenvironment) originates from a heterogeneous group of diseases (i.e., chemical or thermal injuries, Stevens-Johnson syndrome, microbial infections, aniridia, keratitis, and chronic limbitis), in which the LCSs are unable to remodel the corneal epithelium due to the scarce number of cells and excessive limbus damage. This serious condition requires a specific therapeutic approach to treat such patients; it can thus be concluded that the limbus and the LSC functions are of paramount importance [7,8,9]. Reported surgical techniques involved in the LSCs deficiency treatment include amniotic membrane (AM) grafting, conjunctival limbal autografting, cultivated and simple limbal epithelial transplantation, cultivated oral epithelial mucosal transplantation, implantation of keratoprosthesis [10]. When using autografts, usually in the case of one eye injury, the healthy eye might develop the same LSC deficiency condition, while in the case of allografts transplantation, the result is dependent on the donor’s organ [11,12]. Some of the limitations in the use of natural membranes, such as the AM, include poor stability in their composition due to donor variations, infections, and scarce availability. Therefore, there are no predictable outcomes after its application [13,14]. For this reason, many researchers are suggesting alternatives such as bio-based synthetic scaffolds, one of which are electrospun scaffolds. Electrospinning is the process of forming nanofibers with the aid of a high-voltage power supply that stretches a viscoelastic polymer solution or melts [15]. The solidification mechanism of the fibers in solution electrospinning occurs through solvent evaporation, while in melt electrospinning, solidification occurs through cooling. The advantages of melt electrospinning over the solution-based technique are higher efficiency and eco-friendliness due to solvent-free production, while the limitations include high viscosity, large fiber diameters, and modeling difficulties [16]. The melt electrospinning [17] technique is generally utilized to a much lesser extent compared to solution electrospinning [18]. Although the biggest disadvantage of melt electrospinning is the limited number of polymers (especially natural ones) that can be employed in scaffold production, the possibilities that this technique offers in terms of 3D structure design counter this limitation. Within this context, melt electrowriting is getting more popular in recent years since it allows the fabrication of scaffolds with well-controlled layers of filaments with target dimensions and densities. Such graded architectures can closely mimic morphologically complex human tissues. This feature of the melt electrowriting technique comes from the combination of melt electrospinning and 3D printing [19]. It was reported that changing the air pressure and collector speed in melt electrowriting without any changes in the electrical voltage allows the manipulation of fiber diameter over one magnitude, and the filaments can be positioned with high accuracy to result in a complex scaffold morphology [20]. Melt electrowriting technique was used for the fabrication of a multilayer, gradient structure of the eye native human trabecular meshwork (HTM), where polycaprolactone (PCL) constructs with fiber diameters of 10–12 μm and thickness of 125–500 μm supported cultured HTM cells viability and morphology [19]. Real-size aortic roots with the sinuses of the Valsalva and a tri-layered fiber architecture were produced by melt electrowriting to support the adhesion of human umbilical vein smooth muscle cells [21]. Similarly, a viable corneal stroma substitute was designed by culturing human keratocytes onto melt electrowritten highly organized fibrous PCL, which resulted in the formation of a new tissue comprising keratocan and collagen I, V, and VI [22]. Corneal stroma replacement was also suggested to be grown onto melt electrowritten PCL with an orthogonal 3D arrangement, showing deposited collagen fibrils of the corneal stromal cells laid entirely within and across the open pores of the scaffold. This confirmed crucial topographical cues for cell support since collagen was laid only in the uniaxial direction of the aligned fibers in the case of solution electrospun scaffolds [23]. A structure similar to the native cornea was also successfully fabricated by 3D printing of a composite ink of gelatin methacrylate modified by hyaluronic acid. Rabbit-derived corneal stromal cells were further incorporated into the printed scaffolds to help modulate the extracellular matrix remodeling [24]. In addition to structural integrity, another challenge in the design of bio-based electrospun scaffolds for the reconstruction of damaged ocular tissues is scaffold transparency. In the case of solution electrospun PCL, authors reported enhanced optical transparency of plasma-treated PCL scaffolds, showing more than 47% higher transmittance at a wavelength of 700 nm in the wet state compared to wet untreated PCL scaffolds [25]. Other authors discussed the fabrication of transparent PCL/collagen scaffolds for the culture of rabbit corneal cells to be used as corneal grafts for ocular tissue reconstruction. The scaffolds had a 3D hemispherical structure with radially aligned nanofibers showing the optical intensity of the cornea of 0.1 in the wavelength from 400 to 800 nm [26]. Generally, in solution electrospinning, if the selection of the materials is excluded, scaffolds obtain transparency with post-processing operations (heat treatment, solution treatment, and surface modification) or certain set-up modifications (target topological fiber arrangements) [27]. The main cause of the light loss in materials is the reflected light, which in the case of solution electrospun scaffolds increases due to the high interface area resulting from the large number of interconnected pores, which finally results in scaffold opaqueness [27,28,29]. In melt electrowriting, scaffold transparency can be obtained more easily due to the controllable precise deposition of the filaments and the microfibrous structure. In our study, transparent scaffolds were fabricated from polylactic acid (PLA) with a higher melting point and an increased rate of crystallization. PLA is a thermoplastic aliphatic polyester that originates from organic lactic acid, and due to its biodegradability, good biocompatibility, good processability, and food and drug administration approval are, well-known for its applications in the medical fields such as orthopedics tissue engineering, facial fracture repair, ureteral stents, and drug delivery [30]. The transparency of our scaffolds was not lost even when the PLA scaffolds were modified with silk fibroin and gelatin, also well-known biomaterials. Silk fibroin is a natural fibrous protein originating from silkworms and spiders, with remarkable physiochemical (biocompatibility, bioresorbability, biodegradability, and low immunogenicity) and mechanical properties [31,32]. Gelatin is also a natural polymer derived from animal collagen from skins, bones, and tendons, but mainly from porcine skin collagen. Apart from biodegradability and biocompatibility, its unique properties include low antigenicity with no toxic byproducts after degradation, accessibility for chemical functionalization as well as availability and cost-effectiveness [33,34]. Both silk fibroin and gelatin are successfully fabricated into biomedical scaffolds based on solution electrospinning. For example, silk fibroin was combined with PLA for the development of a biomimetic meniscus scaffold [35], or gelatin was blended with chitosan for the in vitro study of human dermal fibroblast cells culture for the application in skin tissue engineering [36]. Other tissue engineering applications include bone [37], nerve [38], vascular [39], and tissue repair. The objective of this study was to develop bio-based, composite melt electrospun (electrowritten) scaffolds from silk fibroin or gelatin-modified microfibrous PLA for the regeneration of ocular tissues. The main role of the scaffold was to support the adhesion of limbal stem cells and their viability, as well as p63 and CK3 expressions to confirm growth, proliferation, and corneal epithelial differentiation.

## 2. Materials and Methods

The polymer used in the current study was polylactic acid (PLA), Luminy^®^ L175—Total Corbion, Lach-Ner Ltd., Zagreb, Croatia. Silk fibroin were used in the form of a silk powder, Huzhou Xintiansi Bio-tech Co., Ltd., Huzhou, China. Pork gelatin was kindly supplied by the Faculty of Food Technology and Biotechnology of the University of Zagreb, Croatia. Other chemicals used for the post-processing treatments and cell cultures were as follows: MgCl_2_/ethanol/ddH_2_O (Sigma-Aldrich, Sant Louis, MO, USA), Dulbecco’s modified eagle medium (DMEM) (Gibco, Paisley, UK), ethylenediaminetetraacetic acid (EDTA) (Sigma-Aldrich, Sant Louis, MO, USA), buffered formaldehyde solution, methanol solution (Sigma-Aldrich, Sant Louis, MO, USA), phosphate-buffered saline (PBS) (Sigma-Aldrich, Sant Louis, MO, USA), antibiotics (penicillin and streptomycin) antifungal drug (amphotericin B) (Gibco, New York, NY, USA), mitomycin C (Sigma-Aldrich, Sant Louis, MO, USA), dyes trypan blue (Life Technologies, New York, NY, USA) and Coomassie blue staining (Thermo Fisher Scientific, Waltham, MA, USA).

**Figure 1:**
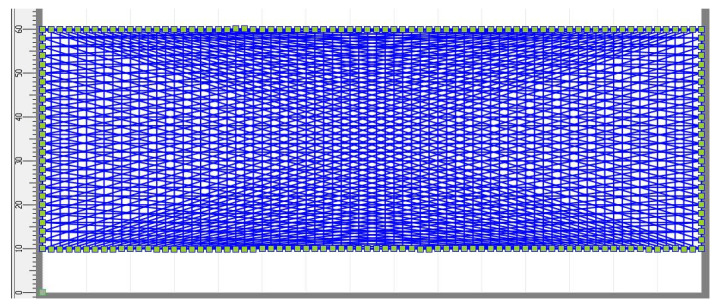
A 2D model of the melt electrospun PLA scaffolds; top view of the first layer; all six layers are identical.

PLA scaffolds were prepared on a melt electrospinning device, Spraybase, AVECTAS, Maynooth University, Co. Kildare, Ireland. The processing parameters were as follows: working temperature 200 °C, heat to collector distance 1.2 cm, air pressure 1.3 bars, and electrical voltage 11.5 kV. The scaffolds were electrospun based on a previously designed 2D model, Figure 1, in the device’s corresponding software SEL generator. The 2D model represents a net consisting of vertically, horizontally, and diagonally (angle smaller than 90°) distributed filaments. The electrospinning was conducted in 6 layers, generating a thickness of 1.222 ± 0.181 mm (6 measuring points), while the scaffold dimensions were 50 × 150 mm. The scaffold’s thickness was measured using Digi Micrometer Mitutoyo, Mitutoyo, Aurora, IL, USA. The scaffolds were imaged with an optic microscope Dino Capture 2.0, Dunwell Tech, Inc., Torrance, CA, USA.

### 2.1. Post-Processing of Melt Electrospun PLA Scaffolds

The melt electrospun PLA scaffolds were cut into 14 mm disks with a metal cutter. The scaffolds were further immersed in a sterile physiological solution and left overnight. The next day, the disks were incubated for 6 h in a concentrated antibiotic and antimycotic solution and washed again in a sterile physiological solution. These scaffolds were used as controls.

**Figure 2:**
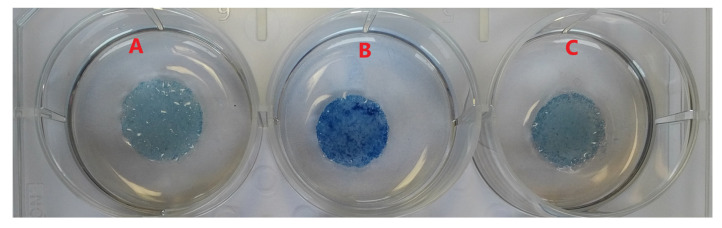
Coomassie blue staining: (**A**) PLA, (**B**) PLA/silk fibroin, and (**C**) PLA/gelatin scaffold.

Next, the hydrated PLA disks were transferred to a sterile 24-well cell culture plate (TPP—Techno Plastic Products AG, Trasadingen, Switzerland). The silk fibroin or gelatin coatings on the PLA scaffolds were confirmed by Coomassie blue staining solution, as shown in Figure 2.

PLA scaffolds were additionally coated with silk fibroin or gelatin by pre-incubation in a 2% solution of silk fibroin in MgCl_2_/ethanol/ddH_2_O (0.8/2/8 molar ratio) or a 10% solution of pork gelatin in dH_2_O overnight at room temperature. The scaffolds were further washed in ddH_2_O and incubated in concentrated antibiotic and antimycotic solution (500 IU penicillin, 500 μg streptomycin, 1.25 μg amphotericin B per mL).

### 2.2. PLA Feeder Cells and Limbal Stem Cells (LSCs) Culture

LSCs were expanded from three different donors stored in liquid nitrogen in passages 1, 4, and 4. Thawed LSCs were expanded on mitomycin C (10 μg/mL) treated 3T3 cell monolayer, reaching 80% confluence. Before the LSCs seeding, DMEM supplemented with 10% fetal bovine serum (Life Technologies, New York, NY, USA) was removed from 3T3 feeder cells and replaced with a LSCs re-suspended medium. The PLA scaffolds were seeded with 70.000 mitomycin treated 3T3 cells in passage 8 and subsequently with 140.000 LSCs in 0.5 mL of the LSCs medium. The culture lasted for a total of 5 days, while the LSCs nutrient medium was replaced every two days.

### 2.3. Scanning Electron Microscopy Analysis

The LSCs cultured melt electrospun scaffold morphology was examined using a scanning electron microscope (at an accelerating voltage of 5 kV) SEM-FE MIRA II LMU, TESCAN, Brno–Kohoutovice, Czech Republic, at the University of Zagreb Faculty of Textile Technology, Department of Textile Chemistry and Ecology. Prior to the imaging, the samples were treated according to the procedure given elsewhere [40] in order to fixate the cultured cells.

### 2.4. Total Porosity Calculation, Fiber Diameter, and Pore Area Measurement

The total porosity of the scaffolds was calculated according to the equation given elsewhere [41], thus, based on their thickness, area, weight, and density of the polymer and the blend. The thickness of the scaffolds was measured by a Digi Micrometer, Mitutoyo, and the calculation of the porosities was carried out in triplicates. The scaffold’s fiber diameter and pore area were determined based on the optical Dino Capture 2.0 microscope images by measuring 30 or 100 randomly selected fibers or pores, respectively, using the ImageJ-NIH software.

### 2.5. Biodegradation of the Melt Electrospun Scaffolds

The biodegradation of the electrospun scaffolds was evaluated by incubating the scaffolds in a standard saline solution of 0.9% for 7 days at a temperature of 37 °C. The samples were cut into 2 × 2 cm pieces, immersed in 40 mL of the solution, and stirred continuously at 160 rpm. The saline solution simulates the natural environment of the limbal stem cells in the corneal tissue. The biodegradability was determined based on the scaffolds’ weight loss on days 3 and 7, on which the samples were taken out from the solution, dried, and weighed. The weight loss ($W_{l}$) was calculated based on the initial dry weight ($W_{dry1}$) and the dry weight ($W_{dry2}$) after incubation, by Equation (1) [42]:

(1) $$W_{l}=\frac{W_{dry1}-w_{dry2}}{w_{dry1}}\cdot 100\;\left(\%\right)$$

### 2.6. Absorption Ability of the Melt Electrospun Scaffolds

The absorption ability of the electrospun scaffolds was determined when removing the samples from the standard saline solution on days 3 and 7. The samples were weighed immediately after removing the fluid on their surface with filter paper. The swelling percentage $S_{w}$ was calculated using Equation (2) [43], where $W_{dry}$ is the initial dry weight of the samples and $W_{wet}$ is the weight after sample removal from the solution.

(2) $$S_{w}=\frac{w_{wet}-w_{dry}}{w_{dry}}\cdot 100\;\left(\%\right)$$

### 2.7. Scaffold Transparency Evaluation

The scaffold transparency was evaluated by observing an illuminated target (colored letters/text) through the samples. The transparency was expressed as the maximum distance at which the target could be clearly resolved through the material.

### 2.8. Determining the Number of Viable LSCs Adhered to the Single PLA Scaffolds

After five days of LSCs cultivation on melt electrospun PLA scaffolds, the scaffolds were washed in PBS and treated with trypsin in ethylenediaminetetraacetic acid (EDTA) to form the cell suspension. The number of LSCs and viability were determined using 0.2% trypan blue staining and expressed for three independent samples.

### 2.9. Immunodetection of p63α and Cytokeratin 3 in the LSCs Using Flow Cytometry

For the detection of p63α and cytokeratin 3 (CK3), fluorescent conjugated monoclonal antibodies were used after removing the cells from the scaffolds by the treatment with 0.25% trypsin/EDTA in PBS medium. The number of viable LSCs adhered to the single PLA scaffolds (after rinsing in the LSCs medium) was determined in three independent experiments in which three different donors’ LSCs were used (0.2% trypan blue staining). After removal from the scaffolds, the proportion of the 3T3 feeder cells in the LSCs suspension was determined by labeling the cells with an anti-feeder antibody (clone mEF-SK4) that specifically binds the antigen expressed on the 3T3 cells only.

For the immunodetection of p63α and cytokeratin 3, the LSC cells were fixed in a 4% buffered formaldehyde solution and permeabilized in a 90% methanol solution. After washing, the cells were labeled with monoclonal antibodies for p63α (clone C-12) or cytokeratin 3 (clone AE5) and analyzed with a Becton Dickinson LSR II flow cytometer.

### 2.10. The Detection of LSCs Adhered Onto the Modified PLA Scaffolds

Incucyte^®^ Nuclight Rapid Red Dye for Live-Cell Nuclear Labeling (dilution 1:500) was used to visualize the cells on the scaffolds. This dye specifically binds to the DNA of the cells and enables “real-time” monitoring of cell growth with minimal cytotoxicity. The fluorescence of the labeled LSC before seeding was analyzed on the EVOS Image Station (Thermo Fisher Scientific, Waltham, MA, USA) and ImageXpress Micro Confocal High-Content Imaging System (Molecular Devices, San Jose, CA, USA). For confocal microscopy, images were taken in a Z-stack of 42 µm with a 3 µm distance between the planes. Geometry was IXConfocal module disk 60 µm pinhole. The objective used was Nikon 20× Ph1 S Plan Fluor ELWD (Nikon, Japan).

### 2.11. Statistical Analysis

The results concerning fiber diameter, porosity, swelling percentage, and weight loss are given as means ± standard deviations. Analysis of variance (ANOVA one-way) with Tukey post-test for means comparisons was performed in OriginPro to estimate statistical significance at the level of *p* < 0.05. The means were significantly different at this level for the measured fiber diameter, pore area (between PLA and PLA/gelatin), swelling percentages at day 7, the weight loss at both day 3 and day 7, and the viability/number of LSCs.

## 3. Results and Discussion

**Figure 3:**
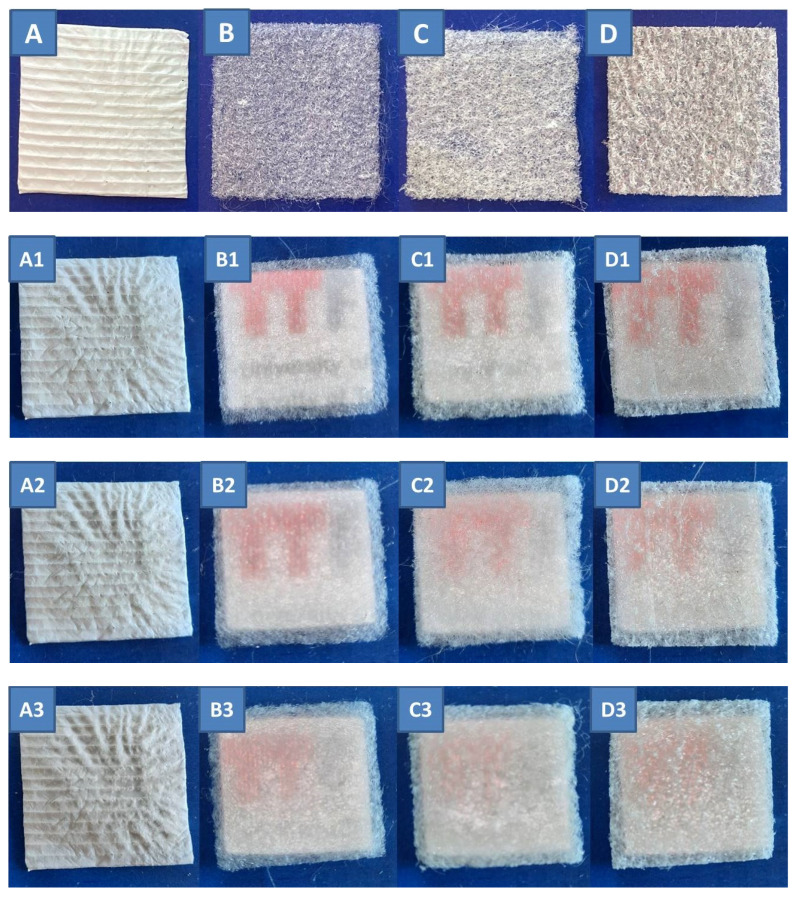

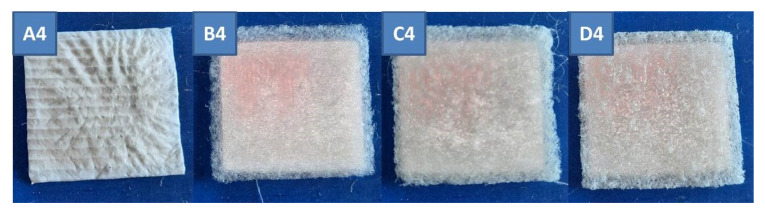
Photographs of: (**A**) PCL electrospun solution and the electrospun melt, (**B**) PLA, (**C**) PLA/silk fibroin, and (**D**) PLA/gelatin scaffolds; sample dimensions 30 × 30 mm; samples on illuminated letter/text background with a distance of (**A1**–**D1**) 0 cm; (**A2**–**D2**) 0.5 cm, (**A3**–**D3**) 0.7 cm and (**A4**–**D4**) 1.2 cm.

Figure 3A–D shows photographs of the electrospun melt, Figure 3B PLA, Figure 3C PLA/silk fibroin and Figure 3D PLA/gelatin scaffolds, and electrospun solution, Figure 3A PCL scaffold for comparison, all on top of a blue non-illuminated background.

Figure 3A1–D1 shows photographs of the same scaffolds positioned on top of an illuminated colored letters/text background at a distance of 0 cm. The distance between the samples and the illuminated background was increased as follows: Figure 3A2–D2 at 0.5 cm, Figure 3A3–D3 at 0.7 cm, and Figure 3A4–D4 at 1.2 cm. Comparing the melt electrospun PLA scaffold with the PCL electrospun solution scaffold (given as an example), it is clearly visible that the PCL is a non-transparent material at all distances, while all electrospun melt scaffolds showed transparency at a certain distance.

The maximum distance at which the illuminated target can be relatively clearly resolved was the highest (0.7 cm) for the single PLA scaffold, while the distance was 0.5 cm in the case of the silk fibroin and gelatin-modified PLA scaffolds. This was expected due to the silk fibroin and gelatin coatings on the surface of the PLA scaffolds.

**Figure 4:**
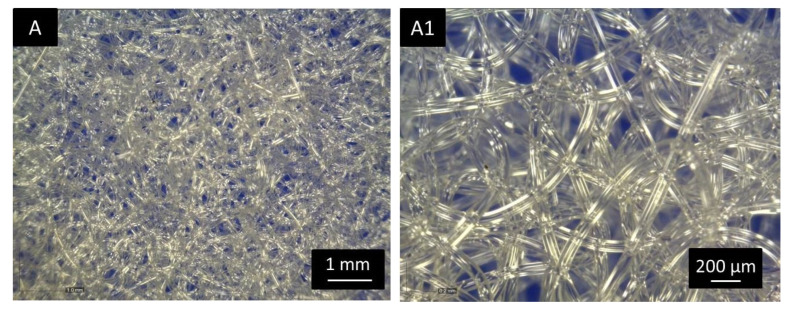

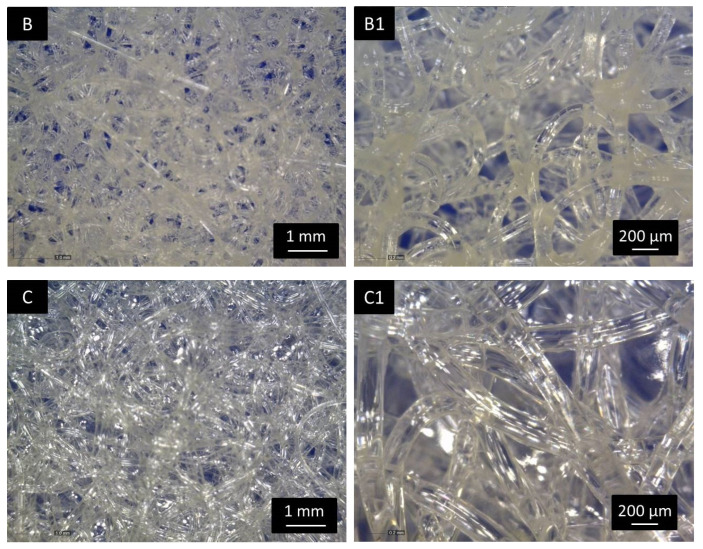
Optical images (magnifications 60× and 200×) of the electrospun melt (**A**,**A1**) PLA, (**B**,**B1**) PLA/silk fibroin, and (**C**,**C1**) PLA/gelatin scaffolds.

The transparency of the single melt electrospun fibers is also shown in Figure 4, which gives the optical images of the PLA electrospun melt (Figure 4A,A1) and modified PLA/silk fibroin (Figure 4B,B1) and PLA/gelatin (Figure 4C,C1) scaffolds at 60× and 200× magnifications. The images show the micro random architecture, although the filaments were spun according to the 2D model geometry on the macro level, as shown in Figure 1. The 3D micro-mesh structure was the result of the loop formation along the PLA filaments (positioned in compliance with the given geometry) during electrospinning caused by the instability of the polymer melting jet.

Similarly, studies concerning melt electrowriting of PLA reported difficulties in the accurate positioning of the PLA fibers due to high PLA viscosity [44]. Some authors reported the addition of sodium stearate to reduce the melt viscosity of PLA during electrospinning, which also resulted in a reduction of fiber diameter [45].

**Figure 5:**
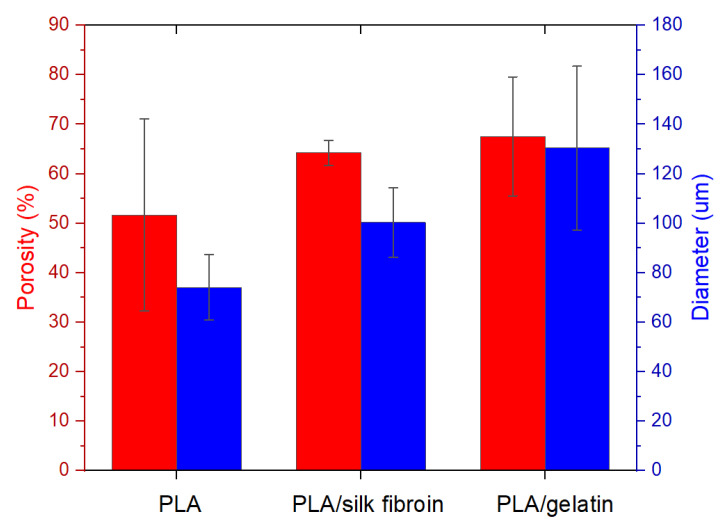
Porosity and average fiber diameter of the electrospun PLA melt PLA and modified PLA scaffolds.

Figure 5 shows the calculated total porosity of the electrospun PLA melt and modified PLA scaffolds, as well as their average measured fiber diameter. Both fiber diameter and total porosity showed an increasing trend from the single PCL to the gelatin-modified electrospun PCL scaffolds. The measured average fiber diameters were 74.17 ± 13.12, 100.33 ± 14.05, 130.43 ± 33.13 µm for the PLA, PLA/silk fibroin, and the PLA/gelatin electrospun scaffolds, respectively. These results were expected since silk fibroin, and gelatin solutions formed a layer/coating on the surface of the PLA filaments after the post-processing treatment. The increase in fiber diameter resulted in the increase (although not with a significant difference) of the scaffold’s total porosity from 51.68 ± 19.39% to 67.56 ± 2.53%, which can be the result of the modification process or the silk fibroin and gelatin solution entering the layers of the scaffold and loosening the compactness of the microfibrous structure. In solution electrospinning, this is usually due to the fact that thicker microfibers are unable to accumulate more compactly on the collector [46]. Some of the reported porosities of the PCL electrowritten melt PCL were between 77.7 and 90.7% [47] or from 84.2 to 91.2% [19].

**Figure 6:**
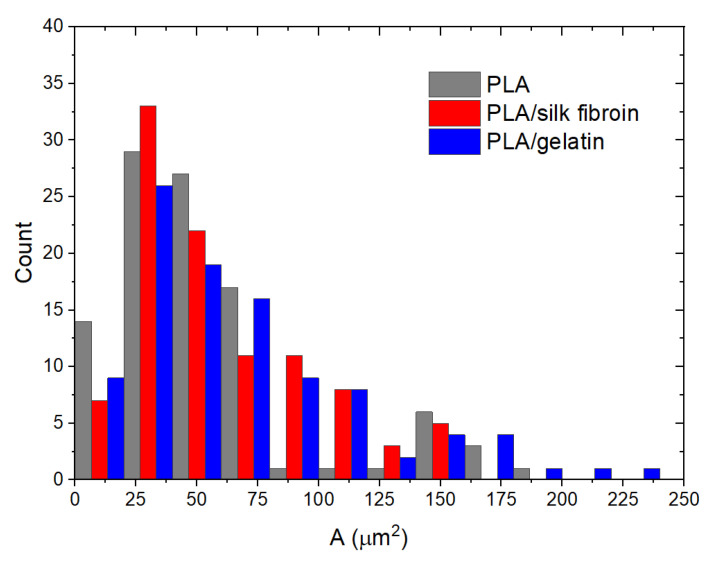
Pore area distribution of the melt electrospun PLA and modified PLA scaffolds.

Figure 6 shows the measured pore area distribution of the melt electrospun PLA and the silk fibroin and gelatin-modified PLA scaffolds. The ranges of the measured values were as follows, 8~200 µm^2^, 7~160 µm^2,^ and 6~230 µm^2^ for the single PLA, PLA/silk fibroin, and PLA/gelatin scaffolds, respectively. The same trend, as in the case of the scaffolds’ porosity and fiber diameter, was observed for the pore area. Thus, there was an increase in the mean values of the pore area from 53.72 ± 41.96, 58.87 ± 36.55 to 68.04 ± 47.68 µm^2^, for the single and the modified PLA scaffolds, respectively. The significant difference calculated was between the PLA and the gelatin-modified PLA scaffolds. It means that in terms of morphology, the gelatin has primarily affected the scaffold’s initial structure, which will further affect cell behavior. Some of the human cells’ reported sizes are between 30 to 100 µm (i.e., pancreatic beta cells, keratinocytes, fibroblasts, and adipocytes) [48], although there are smaller and bigger cell types as well. The measured sizes of the limbal and corneal basal cells were 10 and ~17 μm, respectively [49]. Generally, our PLA-based scaffolds, in terms of pore area, may fit smaller cell types, or as investigated elsewhere [50], smaller pore sizes (i.e., 50 µm) are more suitable for initial cell adhesion due to better cell support from the scaffold’s structure. Pores that are too large (i.e., 400 µm) may influence cells’ growing time [47], but larger pores are needed for further cell proliferation [50]. The suitability of the pore size depends on the cell type, as different cells have optimal pore sizes [50]. Our scaffolds have a range of distributed pore areas (from smaller to larger), which may also be beneficial for cells’ initial and further growth.

**Figure 7:**
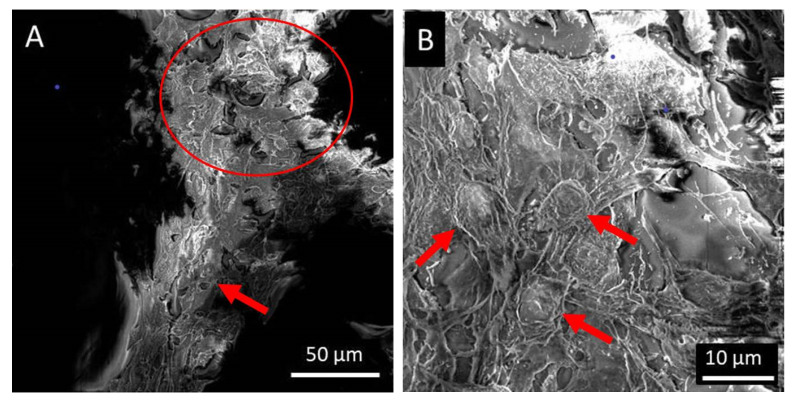

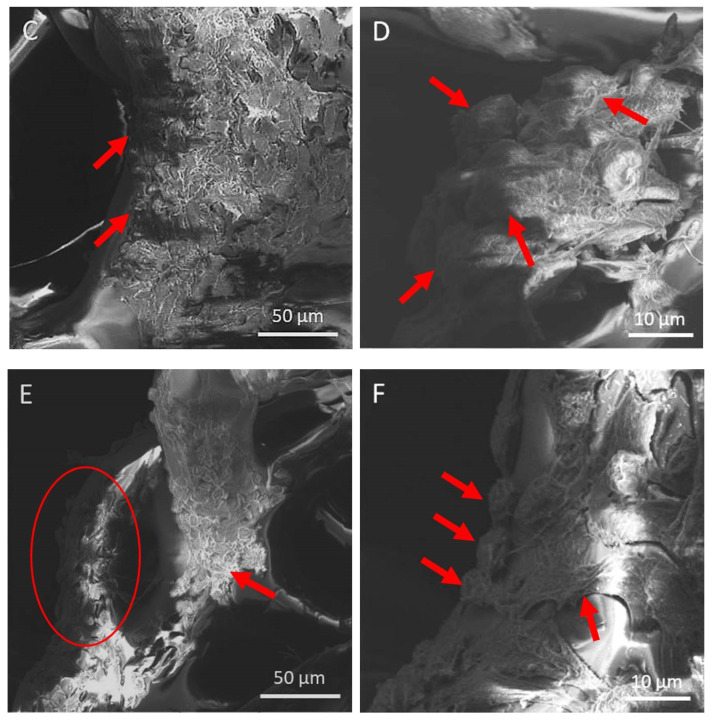
SEM images of cultured LSCs: (**A**,**B**) PLA, (**C**,**D**) PLA/silk fibroin, and (**E**,**F**) PLA/gelatin scaffolds.

Figure 7 shows the SEM images of the single electrospun PLA melt, PLA/silk fibroin, and PLA/gelatin scaffolds. Generally, all scaffolds confirmed the adhesion of the LSCs all around the PLA or silk fibroin and gelatin-coated PLA micro filament surfaces. On top of the fiber surfaces, the LSCs showed flat or elongated morphologies (Figure 7A,C,E), while side views of the filaments also revealed somewhat 3D or spherical structures (Figure 7B,D,F).

**Figure 8:**
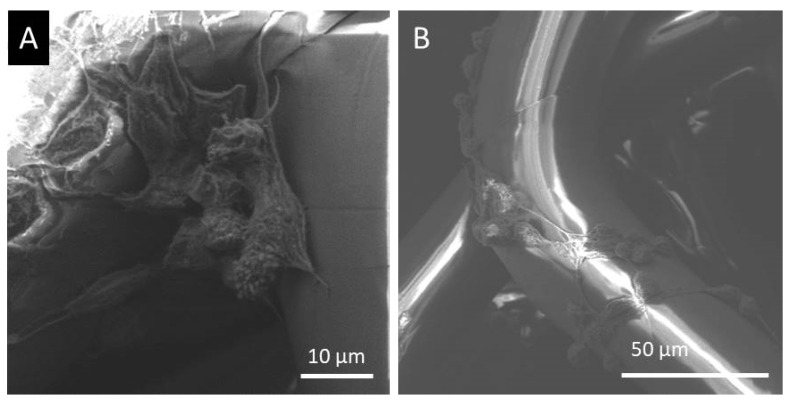
SEM images of the spherical structure of the LSCs cultured on the PLA/gelatin scaffolds, the (**A**) scale of 10 µm and (**B**) scale of 50 µm.

The cells seemed to be firmly attached to the surfaces of microfibers, and the LSCs showed the formation of colonies in flattened larger conformations. The side views of the microfibers also revealed 3D spherical LSCs agglomerations (Figure 8A), but the cells were also forming bead-on-string conformations around the filament surfaces (Figure 8B). Similarly, sphere-shaped single or numerous LSCs were observed on electrospun PCL or PCL-gelatin scaffolds [14].

**Figure 9:**
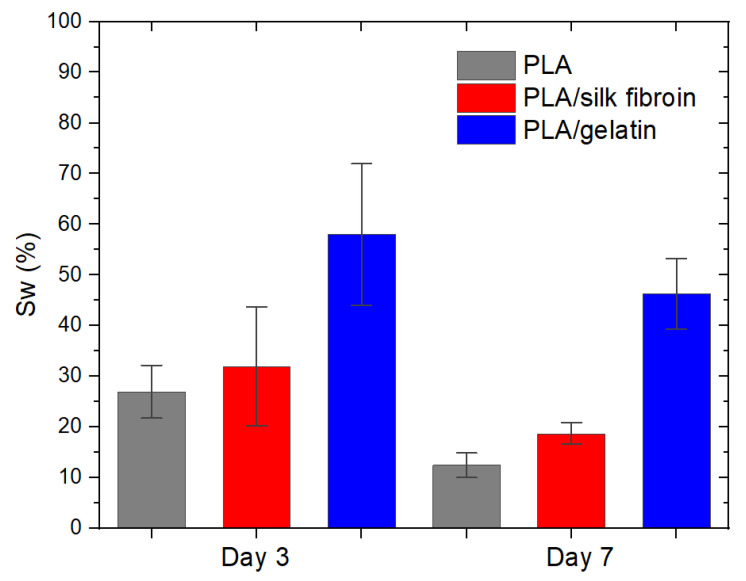
Swelling percentage of the electrospun PLA and modified PLA scaffolds on day 3 and day 7 after incubation in saline solution.

In the design of an ideal tissue engineering scaffold, one needs to consider the suitable fluid absorption ability of the material since extremely high water uptake will destroy its structure, while lack of it will inhibit cell growth [51]. The absorption ability of the scaffold is represented by its swelling percentage Sw (%), directly related to its hydrophilicity or the material’s water content, which equals up to 80% in the corneal tissue [52,53,54]. On the other hand, the biodegradability of the scaffold is extremely important for new tissue generation, and it must maintain adequate mechanical integrity for proper damaged tissue recovery. The degradation rate should also be optimal and meet the extracellular matrix cells’ secretion in order for the scaffold to be replaced by the new tissue [55,56]. In our study, the absorption ability of the scaffolds increased with the modification of the melt electrospun PLA with silk fibroin and gelatin-coating (Figure 9). The same trend was observed for both days 3 and 7 after incubation. The highest Sw was calculated for the PLA/gelatin scaffolds, and it was almost 60% on day 3 (the increase was from 26.98 ± 10.45% to 58.00 ± 27.91%. After 24 h, the scaffolds retained their structure, with no signs of PLA hydrolysis. Similar results were reported in the case of PLA/hydroxyl apatite (HA) scaffolds, where the HA drastically improved water absorption from 65.2 to 159.3% [51]. In the current study, the scaffolds showed reduced swelling percentage at day 7, which can be explained by the gradual removal of silk fibroin and gelatin in saline solution. Since PLA is insoluble in water, biodegradation is very low, below 5% on days 3 and 7 after incubation.

**Figure 10:**
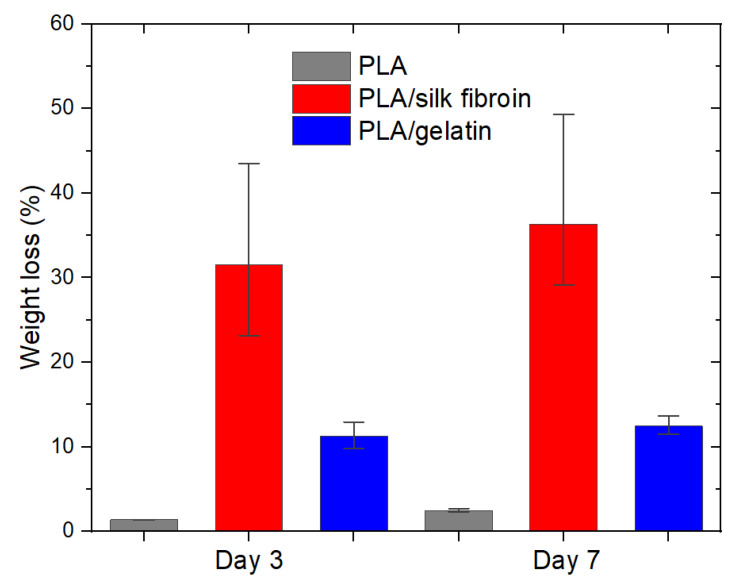
Biodegradation of electrospun PLA and modified PLA scaffolds on day 3 and day 7 after incubation in saline solution.

Both PLA/silk fibroin and PLA/gelatin scaffolds showed a considerable increase in weight loss compared to the single PLA, which was up to 36.31 ± 11.29% and 12.49 ± 1.10% on day 7, respectively (Figure 10). The results were expected since silk fibroin powder is partially soluble in water, and its solubility increases with the increase in temperature, while gelatin is soluble in hot water.

The gradual increase of the scaffolds’ weight loss % with time is in compliance with similar studies that showed that the biodegradation ability of PLA scaffolds is less than 0.5% after 7 days of incubation [57], or less than 10% after 25 days of incubation [43]. Similar to the Sw (discussed earlier), the biodegradation rate of PLA increased with the addition of a hydrophilic component, i.e., the HA [57].

The electrospun melt PLA and modified PLA scaffolds were further evaluated for their role in supporting the viability and growth of limbal stem cells. The number of LSC cells and viability were determined using trypan blue staining and expressed for 3 independent samples (Table 1). All samples showed at least 98% of viable cells, ranging from 1.05–2.9 × 10^6^ cells for each individual PLA scaffold, thus confirming that both single and modified PLA scaffolds support the growth of the LSCs. The difference between the unmodified and silk fibroin and gelatin-coated PLA scaffolds in a cell yield could be observed, with twice as many cells stripped in comparison to uncoated PLA scaffolds (Table 1). The highest number of living cells was actually identified for the PLA/silk fibroin scaffolds, which is supported by the fact that silk fibroin is the most extensively studied material for cornea engineering and is a proven carrier for both corneal epithelial and limbal cells [58].

**Figure 11:**
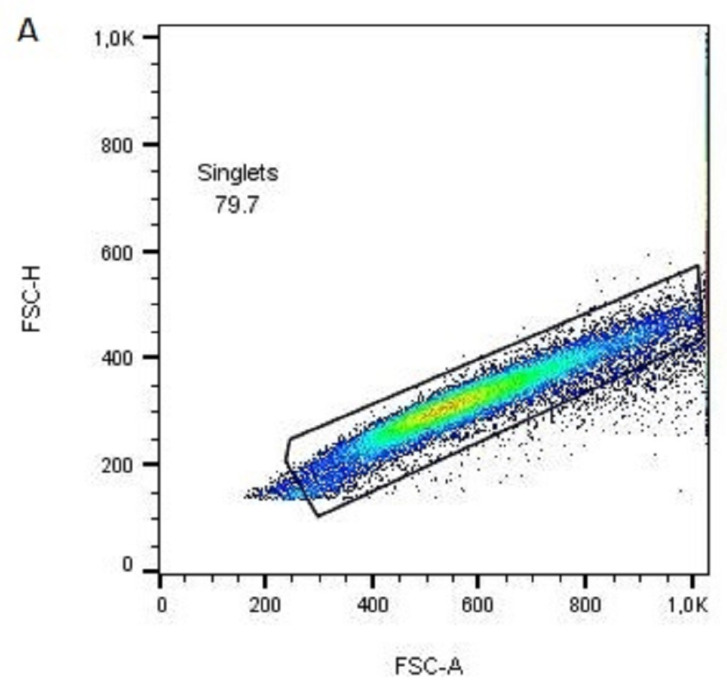

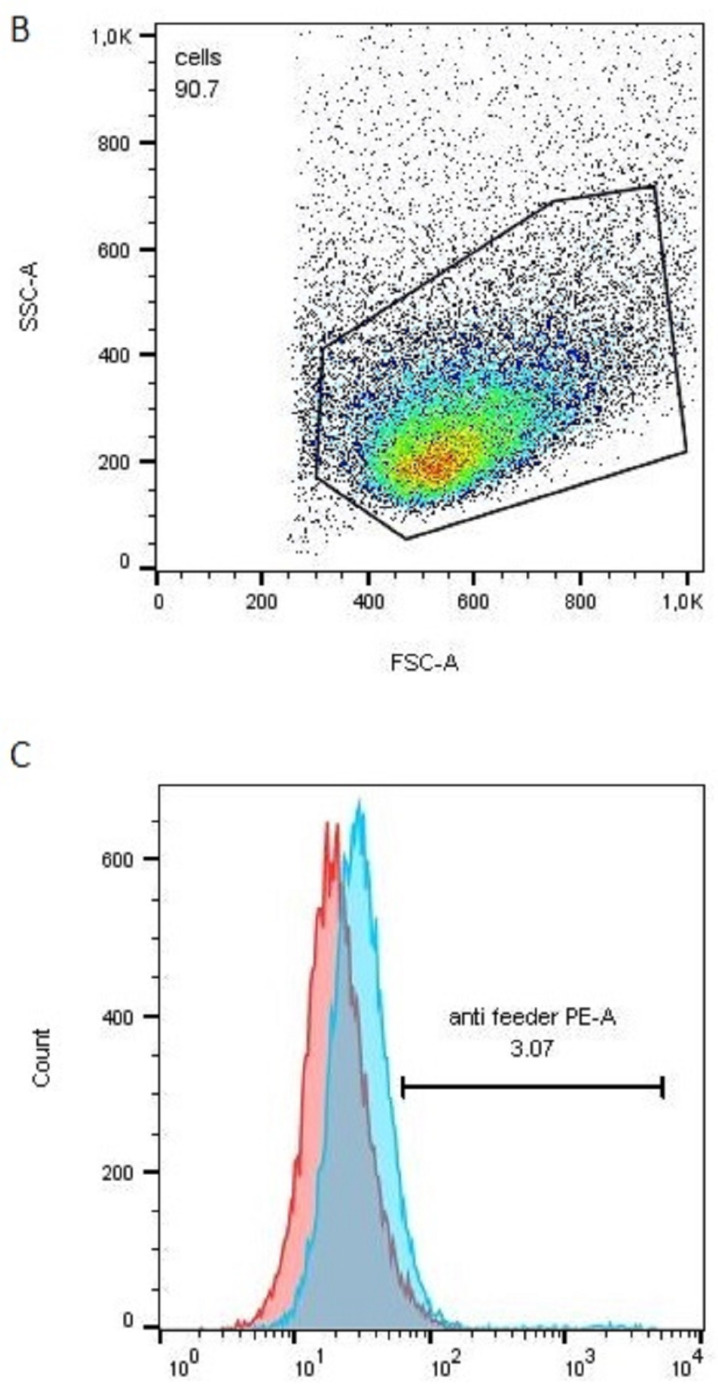
Flow cytometric representative analysis of the proportion of 3T3 feeder cells after removal from the unmodified PLA scaffold (sample 1); (**A**) 79.9% of acquired events in the gate 1 are singlets based on (forward light scatter-area vs. forward light scatter-height) FSC-A vs. FSC-H signal distribution; (**B**) singlets from the gate 1 were analyzed using FSC-A and (sideward light scatter-area) SSC-A signals and individual cells are gated as gate 2 (90.9% of events in gate 1); (**C**) histogram of cells from the gate 2 was analyzed for PE fluorescence for isotype PE-conjugated antibody (red line) or specific anti-feeder cells PE-conjugated antibody (blue line). The limit of the negative/positive signals for feeder cells was set at 1% on a sample labeled with an isotype antibody.

To ensure that counted cells are LSCs, but not proliferating feeder 3T3 cells, stripped cells from different PLA scaffolds were stained with anti-feeder PE-conjugated antibodies to inspect the percentage of live 3T3 cells. Cells were acquired on a flow cytometer, and after excluding doublets, debris, and dead cells (Figure 11A,B), the live cell population was assessed for 3T3 cell frequency (Figure 11C). All cell samples from different PLA scaffolds contained less than 5% of 3T3 cells, which is the recommendation for limbal cell graft transplantation [59].

**Figure 12:**
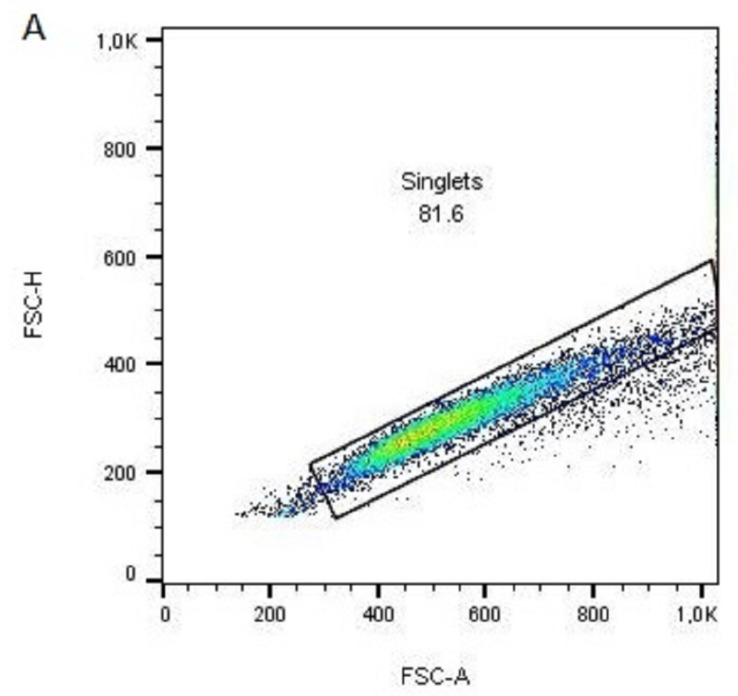

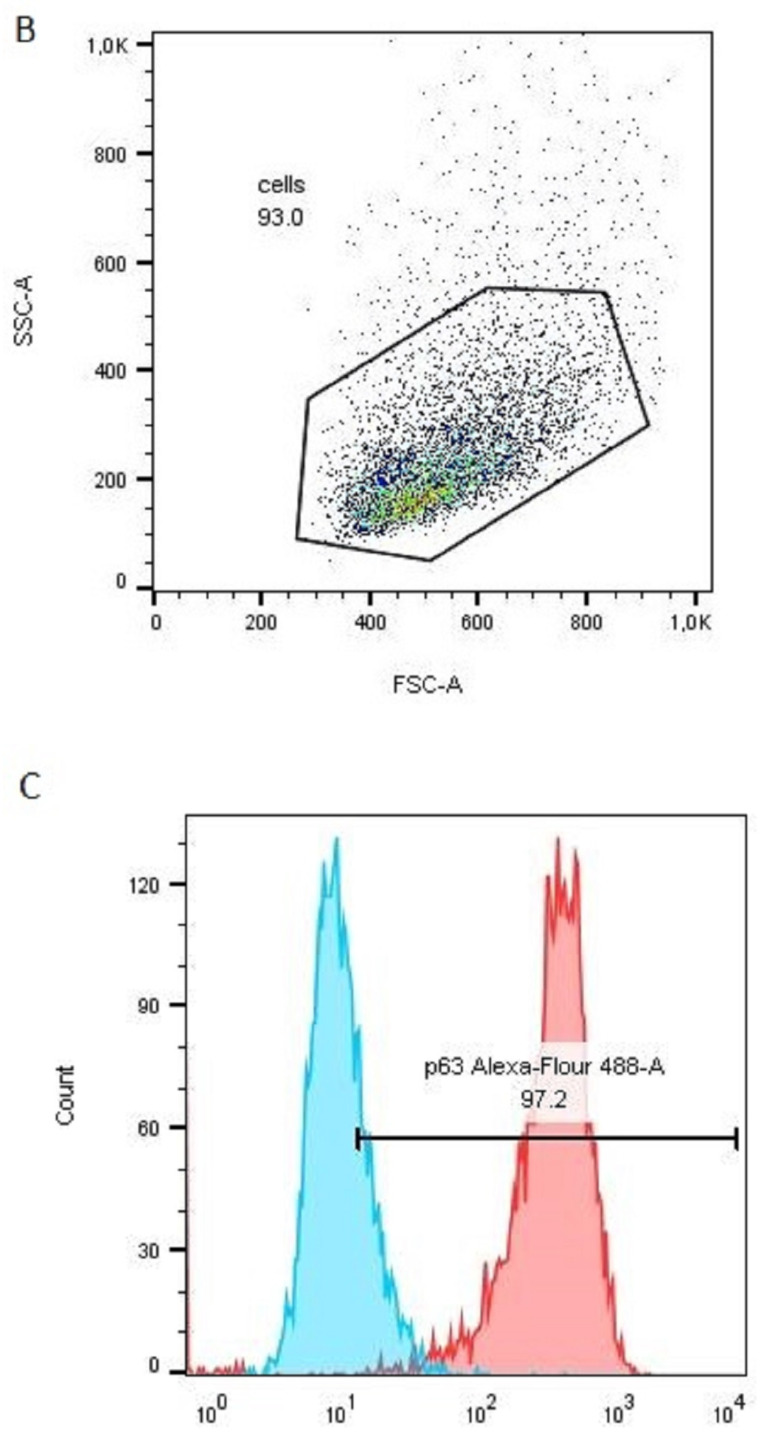

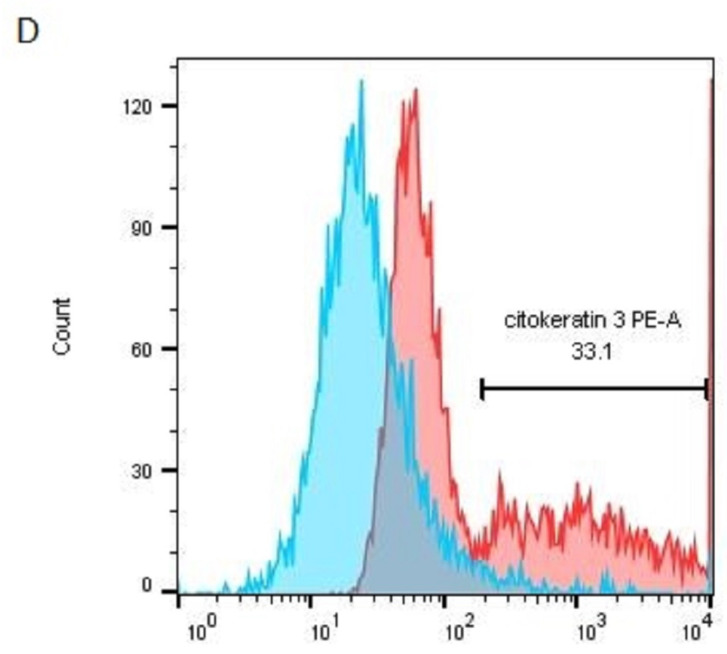
Flow cytometric representative analysis of p63 and cytokeratin 3 expressions in LSC cells stripped from the unmodified PLA scaffold (sample 1); (**A**) 87.7% of acquired events in the gate 1 are singlets based on FSC-A vs. FSC-H signal distribution; (**B**) singlets from the gate 1 were analyzed using FSC-A, and SSC-A signals and individual cells are gated as gate 2 (92.9% of events in gate 1); (**C**) representative histogram of cells from the gate 2 was analyzed for PE fluorescence for isotype PE-conjugated antibody (red line) or specific anti-p63 PE-conjugated antibody (blue line); (**D**) representative histogram of cells from the gate 2 was analyzed for PE fluorescence for isotype PE-conjugated antibody (red line) or specific anti-CK3 PE-conjugated antibody (blue line); The limit of the negative/positive cells was set at 1% on samples labeled with isotype antibody for both histograms.

The assessment of the LSC stemness and differentiation capacity of the cells growing on different PLA scaffolds was analyzed by counting the p63- and CK3-expressing cells on the flow cytometer. Cells were stripped from the PLA scaffolds and stained with the anti-p63 Alexa Fluor 488 or anti-CK3 PE antibodies. Figure 12 shows the representative graphs of gating single and live cells (Figure 12A,B), as well as the representative histograms showing the percentages of the p63-positive (Figure 12C) and CK3-positive (Figure 12D) LSCs. A study reported long-term corneal regeneration (in 78% of patients) after transplantation of cells from cultures in which more than 3% of the total number of clonogenic cells are p63-bright cells [60]. LSC holoclones are thus characterized by high p63 expression, particularly ΔNp63α isoform, which guarantees successful engraftment of an LSC transplant [60]. On the contrary, terminally differentiated cells express CK3 [61] and could be used to evaluate the differentiation potential of the LSCs.

The single-cell analysis confirmed that all three types of PLA scaffolds maintain a high stem potential of proliferating LSCs (based on high p63 expression), as well as low differentiation status of LSCs (based on low CK3 expression) necessary for successful corneal regeneration upon transplantation (Table 2).

Melt electrospun microfibrous scaffolds form a 3D mesh porous structure with the ability to bind cells and form a monolayer on single PLA scaffolds, as well as silk fibroin and gelatin-modified PLA scaffolds.

**Figure 13:**
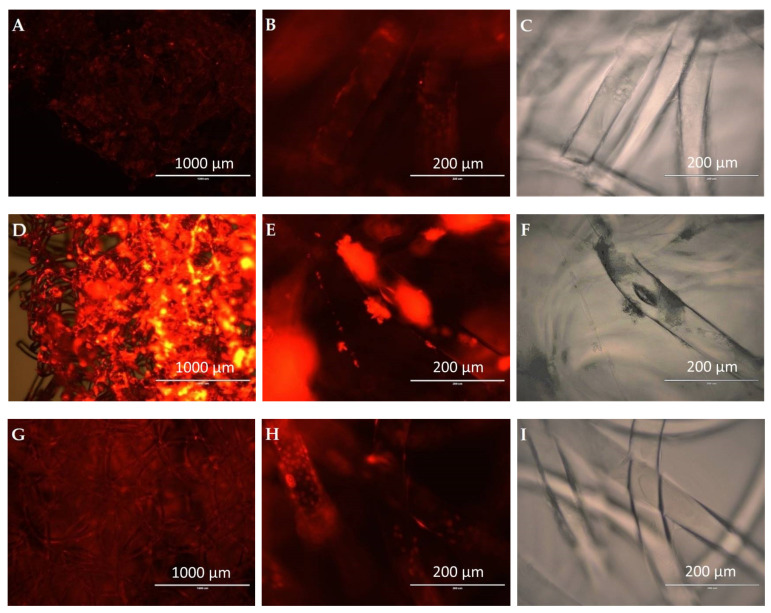
Melt electrospun scaffolds cultured with LSCs stained with Incucyte^®^ Nuclight Rapid Red fluorescent dye: (**A**) single PLA (magnification 4×), (**B**) single PLA (magnification 20×), (**C**) bright field image of single PLA (magnification 20×), (**D**) PLA/silk fibroin (magnification 4×), (**E**) PLA/silk fibroin (magnification 20×), (**F**) bright field image of PLA/silk fibroin (magnification 20×), (**G**) PLA/gelatin (magnification 4×), (**H**) PLA/gelatin (magnification 20×) and (**I**) bright field image of PLA/gelatin (magnification 20×). Detection (531/40 nm excitation; 593/40 nm emission).

In Figure 13A,D,G (magnification 4×), it was possible to detect red fluorescence of Incucyte^®^ Nuclight Rapid Red dye (531/40 nm excitation; 593/40 nm emission) stained cells which bind to the cellular DNA. The most intense red fluorescence was observed on silk fibroin-modified PLA scaffolds (Figure 13D,E), followed by gelatin-modified PLA scaffold (Figure 13G), and finally, the single melt electrospun PLA scaffold (Figure 13A) on EVOS Image Station. The analysis at 20× magnification of the single and gelatin-coated PLA scaffolds did not show the formation of a homogeneous cell monolayer covering the surface of the entire scaffold, but the cells were attached to the individual microfibers that form the surface layer (Figure 13B,H).

These observations correlate with the number of cells adhered onto different PLA scaffolds (Table 1), confirming that the additional silk fibroin and the gelatin coating of the PLA significantly increase LSCs adhesion. The bright field images in Figure 12C,F,I show the transparency of a single melt electrospun PLA, PLA/silk fibroin, and PLA/gelatin microfiber, respectively.

Due to the partial hydrophobicity of PLA, it has been shown that additional processing (modification of the PLA) would enhance the biological properties of PLA scaffolds regarding cell adhesion, proliferation, and differentiation [62]. Coating of PLA with collagen I and Matrigel has proven to enhance cell proliferation [63], whereas PLA 3D scaffolds coated with gelatin, combined with different concentrations of mucic acid, supported not only growth but also differentiation of mesenchymal stem cells into osteoblasts, suggesting beneficial use of modified PLA scaffolds for bone tissue engineering [64]. Similar reported results for the application of PLA in bone tissue engineering concern modifications of PLA with gelatin/magnesium-doped nano-hydroxyapatite [65] or PLA with PCL/gelatin containing different concentrations of ascorbic acid [66]. Although PLA is a relatively rigid material optimal for bone engraftment, it also has been studied for soft tissue engineering due to its high biocompatibility and low toxicity [67]. Gelatin-modified 3D PLA scaffold supported the growth of murine fibroblasts in vitro, and when applied to the lesion, it accelerated complete skin healing [68]. Different biocompatible membranes have been studied as carriers of limbal tissue explants for the treatment of limbal stem cell deficiency, some of which include solution electrospun poly D, L-lactide-co-glycolide (PLGA) to support the growth of isolated limbal epithelial cells (LECs) and the outgrowth of cells from intact limbal explants [69], PLGA for the transfer of LECs to ex vivo rabbit cornea models and five human patients [70], electrospun solution poly (3-hydroxybutyrate-co-3-hydroxyvalerate) (PHBV)/gelatin to replace human amniotic membrane [71], electrospun solution PCL combined with TiO_2_ or cefuroxime to support the growth of limbal stem cells [72], etc. Still, to the best of our knowledge, this is the first study of untreated 3D melt electrospun PLA and silk fibroin or gelatin-coated scaffolds supporting the growth of LSCs in vitro.

In the case of silk fibroin-coated PLA scaffolds, it is possible to observe dense cell agglomerates adhered to silk fibroin complexes. Silk fibroin did not uniformly coat the scaffolds microfibers (Figure 13D), as in the case of the gelatin-coated scaffolds, which implies low PLA surface coating efficiency with silk fibroin. Instead, silk fibroin polymer spontaneously formed higher molecular complexes that localize between PLA microfibers. This has also been observed in a study [35] where directly electrospun PLA/silk fibroin nanofibrous scaffolds confirmed the intrinsic ability of silk fibroin to form complexes. In Supplemental AVI Files (Video S1a–c), 3D fluorescent analysis of PLA scaffolds at 20× magnification, in 100 µm depth of scan, clearly shows the different densities and localization of LSCs related to PLA modifications.

## 4. Conclusions

Melt electrospun (electrowritten) scaffolds seem promising candidates for the regeneration of damaged cornea caused by LSCD. PLA is a biocompatible material of low toxicity, with physical and chemical properties that can be easily altered, but it can reduce cell adherence due to its hydrophobicity. Additional processing, such as chemical modifications or coatings, has been shown to change the biological properties of PLA. In the current study, silk fibroin and gelatin-modified PLA scaffolds provided transparency, larger pore area (68.04 ± 47.68 µm^2^ for the PLA/gelatin), slightly increased porosity (up to 67.56 ± 2.53% for the PLA/gelatin), and improved PLA’s absorption ability (up to 46.34 ± 13.89% for the PLA/gelatin) and biodegradation rate (weight loss %), which was calculated to be the highest (36.31 ± 11.29%) for silk fibroin-modified scaffolds, 7 days after incubation in saline solution. In terms of biocompatibility, both single PLA and silk fibroin and gelatin-modified PLA scaffolds supported the cell adhesion of the LSCs. The SEM images revealed both flat limbal stem cells (LSCs) colonies and 3D spherical LSCs agglomerations with a difference in cell adhesion between single and gelatin-modified PLA on the one hand and silk fibroin-modified PLA on the other, as observed with confocal microscopy. In the first case (PLA and PLA/gelatin scaffolds), the cells adhered directly to the surface of individual microfibers, whereas silk fibroin coating (showing the most intense red fluorescence of Incucyte^®^ Nuclight Rapid Red Dye stained cells) led to the formation of cell agglomerates between microfibers.

The trypan blue staining confirmed at least 98% of viable cells, ranging from 1.05–2.9 × 10^6^ cells adhered onto the PLA scaffolds (highest for the PLA/silk fibroin scaffolds) with the ability to grow, proliferate and differentiate based on the high p63 and low CK3 expressions in the LSCs. Finally, both PLA modifications provided biocompatible substrates for the efficient adhesion and growth of LSCs. However, the application of the gelatin coating resulted in the formation of the surface microfiber layer with a homogenous adherence of the cells along the PLA microfibers, while silk fibroin coating did not form a surface layer, leading to the formation of dense focal cell agglomerates between the microfibers that could represent a bottleneck for further LSCs expansion, as they adhere to uncoated PLA microfibers to a significantly lesser extent.

## Figures and Tables

**Table 1:** Viability and number of LSCs stripped from PLA and modified PLA scaffolds.

Scaffold	LSC Sample 1		LSC Sample 2		LSC Sample 3	
	No. of Cells	% of Cells Viability	No. of Cells	% of Cells Viability	No. of Cells	% of Cells Viability
PLA	1.1 × 10^6^	98	1.2 × 10^6^	99	1.05 × 10^6^	99
PLA/silk fibroin	2.7 × 10^6^	98	2.9 × 10^6^	98	2.2 × 10^6^	99
PLA/gelatin	1.9 × 10^6^	98	2.3 × 10^6^	98	1.75 × 10^6^	99

**Table 2:** Percentages of p63 and CK3 expressions of LSCs grown on PLA and modified PLA scaffolds.

	Scaffold	LSC Sample 1	LSC Sample 2	LSC Sample 3
% p63 + LSCs	PLA	97.3	93.7	73.0
	PLA/silk fibroin	98.7	90.5	71.6
	PLA/gelatin	98.2	92.9	74.7
% CK3 + LSCs	PLA	33.1	41.0	17.2
	PLA/silk fibroin	32.9	42.7	19.8
	PLA/gelatin	35.3	40.1	20.4

## Data Availability

Not applicable.

## Supplementary Materials

The following supporting information can be downloaded at: https://www.mdpi.com/article/10.3390/polym15030777/s1, Video S1a: limbal stem cells adhered onto single melt electrospun PLA scaffold; Video S1b: limbal stem cells adhered onto silk fibroin-modified melt electrospun PLA scaffold; Video S1c: limbal stem cells adhered onto gelatin-modified melt electrospun PLA scaffold.
