# Supplementary material for: Detection of Limbal Stem Cells Adhered to Melt Electrospun Silk Fibroin and Gelatin-Modified Polylactic Acid Scaffolds
Source: Polymers (Basel). 2023 Feb 3;15(3):777. doi: 10.3390/polym15030777 (PMC9919663; doi:10.3390/polym15030777)
Supplement: Supplementary file 1 [file polymers-15-00777-s001.zip › polymers-2097375-supplementary.pdf]

Supplementary material

Detection of limbal stem cells adhered onto melt electrospun – gelatine or silk fibroin modified PLA scaffolds

Emilija Zdraveva[1], Krešo Bendelja[2], Luka Bočkor[3], Tamara Dolenec[4], Budimir Mijović[1]

[1]University of Zagreb Faculty of Textile Technology, Zagreb, Croatia

[2]University of Zagreb, Center for Research and Knowledge Transfer in Biotechnology, Zagreb, Croatia

[3]Institute for Anthropological Research, Zagreb, Croatia

[4]University Hospital Center Sestre Milosrdnice, Department of Transfusion and Regenerative Medicine, Zagreb, Croatia

3D fluorescent analysis of the limbal stem cells detected onto the melt electrospun polylactic acid (PLA) and silk fibroin or gelatine modified PLA scaffolds.

cisti nosac 3D.avi

Video S1a. Limbal stem cells adhered onto single melt electrospun PLA scaffold

silk nosac 3D.avi

Video S1b. Limbal stem cells adhered onto silk fibroin modified melt electrospun PLA scaffold

zelatina nosac 3D.avi

Video S1c. Limbal stem cells adhered onto gelatine modified melt electrospun PLA scaffold
